# Leukotriene Pathway Activation Associates with Poor Glycemic Control and with Cardiovascular Autonomic Neuropathy in Type 1 Diabetes

**DOI:** 10.1155/2020/5704713

**Published:** 2020-03-23

**Authors:** Daniele P. Santos-Bezerra, Luciano R. Filgueiras, Maria Beatriz Monteiro, Sharon N. Admoni, Ricardo V. Perez, Ana M. Cavaleiro, Cleide G. Machado, Ubiratan F. Machado, Marisa Passarelli, Sonia Jancar, Maria Lucia Correa-Giannella

**Affiliations:** ^1^Laboratório de Carboidratos e Radioimunoensaio (LIM-18) do Hospital das Clinicas HCFMUSP, Faculdade de Medicina, Universidade de Sao Paulo, Sao Paulo, SP, Brazil; ^2^Laboratorio de Imunofarmacologia, Departamento de Imunologia, Instituto de Ciencias Biomedicas, Universidade de Sao Paulo, Sao Paulo, SP, Brazil; ^3^Divisão de Oftalmologia do HCFMUSP, Faculdade de Medicina, Universidade de São Paulo, Sao Paulo, SP, Brazil; ^4^Departamento de Fisiologia e Biofisica, Instituto de Ciencias Biomedicas, Universidade de Sao Paulo, Sao Paulo, SP, Brazil; ^5^Laboratório de Lipides (LIM-10) do HCFMUSP, Faculdade de Medicina, Universidade de São Paulo, Sao Paulo, SP, Brazil; ^6^Programa de Pos-Graduação em Medicina, Universidade Nove de Julho (UNINOVE), São Paulo, SP, Brazil

## Abstract

**Background and Aims:**

Since hyperglycemia promotes inflammation by different pathways and inflammation participates in the development of chronic diabetes complications, we investigated the association between the leukotriene (LT) pathway and microvascular diabetes complications.

**Methods and Results:**

Quantitative polymerase chain reaction was employed to quantify the expression of *ALOX5* (encodes 5-lipoxygenase), *LTB4R* (encodes one of the LTB4 receptors), and *MYD88* in peripheral blood mononuclear cells from 164 type 1 diabetes (T1D) individuals presenting or not diabetes kidney disease, retinopathy, peripheral neuropathy, and cardiovascular autonomic neuropathy (CAN); 26 nondiabetic subjects were included as controls. LTB4 plasmatic concentrations were also evaluated. The expression of *LTB4R* was significantly higher in T1D individuals than in controls. T1D individuals with microvascular complications presented lower *MYD88* mRNA expression when compared to those without microvascular complications. Higher LTB4 concentrations were found in individuals with CAN versus without CAN. The observation of two distinct subgroups of T1D individuals in the correlation analyses motivated us to evaluate the characteristics of each one of these groups separately. The group presenting higher expression of *ALOX5* and of *LTB4R* also presented higher values of HbA_1_C, of fructosamine, and of plasmatic LTB4.

**Conclusion:**

In the diabetes setting, the LT pathway is not only activated by hyperglycemia but is also modulated by the status of the autonomic nervous system.

## 1. Introduction

Increasing evidence has accumulated pointing out inflammation as an important player in the development of chronic diabetes complications [1–3]. Hyperglycemia promotes inflammation by distinct pathways, such as oxidative stress-induced activation of NFkB and of NLRP3 inflammasome with subsequent production of proinflammatory cytokines [4]. Additionally, hyperglycemia accelerates the generation of advanced glycation end products (AGEs) that, by binding to RAGE (advanced glycation end product-specific receptor), also activate NFkB in several cell types [5]. This proinflammatory signaling pathway is counteracted by an anti-inflammatory pathway mediated by AGE-R1, a receptor that promotes AGE endocytosis and that acts in synergy with sirtuin-1, exerting anti-inflammatory and antioxidant actions [6].

Bioactive lipid mediators such as eicosanoids can be produced as a consequence of hyperglycemia. A study using mass spectrometry-based metabolomics approach reported changes in the metabolic pathway of eicosanoid synthesis in type 1 diabetes (T1D) individuals. Leukotriene (LT) pathway metabolites were found increased in the blood of T1D individuals after 8 hours of insulin deprivation, which suggests that hyperglycemia could increase concentrations of LT [7]. Moreover, in another study, increased concentrations of LT precursors were found in vitreous samples from diabetes individuals with retinopathy when compared to samples from nondiabetes individuals [1].

LT are produced mainly by leukocytes, although other cell types are able to produce them, following stimuli that activate phospholipase A2. This enzyme cleaves membrane phospholipid-releasing arachidonic acid that can be converted into LTA4 by 5-lipoxygenase (encoded by *ALOX5*). This unstable LT is rapidly converted into LTB4 by LTA4 hydrolase. By interacting with its high affinity receptor, LTB4 potentiates phagocytosis and antimicrobial effector functions in macrophages [8]. Moreover, LTB4 enhances the expression of the adaptor molecule MYD88, amplifying macrophage response to MYD88-dependent stimuli [9]. Since MYD88 is involved in the signaling of IL1 receptor (IL1R), of RAGE, and of almost all toll-like receptors (TLRs), LTB4 enhances the production of cytokines induced by the activation of these receptors, thus potentiating the inflammatory response [10]. In a mouse model of T1D, the low-grade systemic inflammation was characterized by increased concentration of LTB4 and of several proinflammatory cytokines and was significantly attenuated in diabetic mice treated with antagonists of LTB4 [11]. Furthermore, also in this model of T1D, we found that increased susceptibility to sepsis and delayed wound healing were dependent on the high plasma LTB4 concentrations [12, 13].

In the present work, we measured plasma concentrations of LTB4 and analyzed the expressions of *ALOX5*, *LTB4R*, and *MYD88* and of genes related to AGE metabolism in peripheral blood mononuclear cells (PBMC) from long-term T1D individuals, in order to associate them with the presence of diabetes microvascular complications.

## 2. Methods

### 2.1. Participants

One hundred and sixty-four T1D individuals were enrolled in this cross-sectional study (Table 1). All participants were recruited in the Diabetes Outpatient Clinic of Hospital das Clinicas da Faculdade de Medicina da Universidade de São Paulo. Twenty-six nondiabetic subjects were included as the control group (77% women with median (interquartile interval) of 35 (26-55) years old); they did not use statins, angiotensin-converting enzyme inhibitors (ACEI), or angiotensin receptor blockers (ARB). Smokers were not included in this study. The present study was performed in compliance with the Institutional Ethics Committee (Committee approvals #149,940 and #294.169 CEP/CONEP) and the Declaration of Helsinki of 1975, revised in 1983. All participants signed an informed consent.

All T1D participants were evaluated for the presence of the following chronic microvascular complications: diabetic retinopathy (DR), diabetes kidney disease (DKD), peripheral neuropathy, and cardiovascular autonomic neuropathy (CAN), as previously described [14].

### 2.2. mRNA Expression in PBMC

Peripheral blood was collected into BD Vacutainer CPT tubes (BD, Franklin Lakes, NJ, USA) after a 12 h fasting period. The Ficoll method was employed to isolate PBMC, as previously described [15] and plasma was stored at -80°C for further measurements. Total RNA was extracted by the RNeasy Mini Kit (Qiagen, Germantown, MD, USA) after PBMC lysis with TRIzol reagent (Life Technologies, Carlsbad, CA, USA). RNA quantification was performed by NanoDrop (ND-1000 Spectrophotometer), and the integrity of total RNA was assessed by 1% agarose gel electrophoresis. Following the manufacturer's instructions, the High-Capacity cDNA Reverse Transcription Kit (Life Technologies) was employed for reverse transcription of 1,000 ng of total RNA.

Using the StepOnePlus Real-Time PCR System (Life Technologies), mRNA expressions of the following genes were evaluated: *LTB4R*, *ALOX5*, and *MYD88*. Additionally, the expressions of the three genes previously evaluated in this cohort of individuals were correlated with the expressions of *LTB4R*, *ALOX5*, and *MYD88*: *DDOST* (encodes AGE-R1), *AGER* (encodes RAGE), and *SIRT1* (encodes sirtuin-1) [15]. Quantitative PCR was performed as follows: 10 *μ*L of TaqMan Gene Expression Master Mix (Life Technologies), 1 *μ*L of hydrolysis probe set, 10 ng of cDNA, and 7 *μ*L of RNase free H_2_O were mixed. Each sample was run in duplicate. The 2^−*ΔΔ*Ct^ method was used to calculate relative mRNA abundance [16]. As reference, the mean expression of two housekeeping genes was used (*β*-actin (*ACTB*) and *β*2-microglobulin (*B2M*)).

### 2.3. Analysis in Plasma

Plasmatic thiobarbituric acid-reactive substances (TBARS) and reduced glutathione (GSH) were measured in all participants of this study, as previously described [15]. LTB4 was measured by the EIA kit (Cayman Chemical, MI, USA), according to the manufacturer's instructions.

### 2.4. Statistical Analysis

The statistical analyses were conducted with the use of JMP software version 8.0 (SAS Institute, Cary, NC, USA). The mRNA expressions normalized by the reference genes were log_10_ transformed before the analyses. To identify the differences among the studied groups, the nonparametric Wilcoxon signed-rank test followed by Tukey's posttest was employed. Logistic regression analyses were used for adjustment for confounding variables. The correlation analyses were performed by Spearman's rank correlation coefficient. A *P* value of <0.05 was considered statistically significant.

## 3. Results

**Figure 1:**
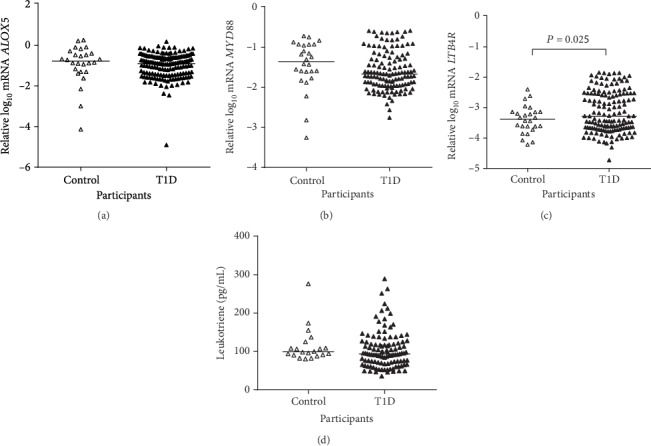
Expressions of *ALOX5*, *MYD88*, and *LTB4R* mRNA in peripheral blood mononuclear cells (a–c) and plasma concentrations of leukotriene B4 (LTB4) (d) in type 1 diabetes (T1D) individuals and in nondiabetic controls (adjusted for sex, age, and use of angiotensin-converting enzyme inhibitor, angiotensin receptor blocker, and statin).

Comparing T1D and nondiabetic controls, no differences were observed in the mRNA expressions of *ALOX5* and *MYD88* and in the plasma concentrations of LTB4 after adjustment for sex, age, and use of statins, ARB, and ACEI (Figures 1(a), 1(b), and 1(d), respectively). The expression of *LTB4R* was significantly higher in T1D individuals (Figure 1(c)).

**Figure 2:**
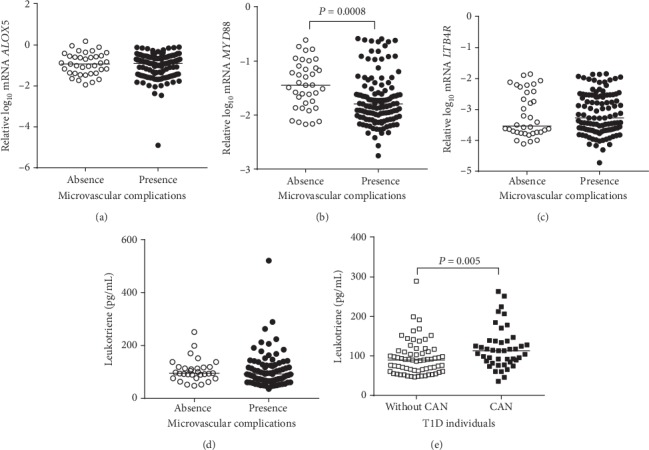
Expressions of *ALOX5*, *MYD88*, and *LTB4R* mRNA in peripheral blood mononuclear cells (a–c) and plasma concentrations of leukotriene B4 (LTB4) (d) in type 1 diabetes (T1D) individuals sorted by the presence or absence of microvascular complications (adjusted for sex, age, HbA_1_C, diabetes duration, and use of angiotensin-converting enzyme inhibitor, angiotensin receptor blocker, and statin). In (e), plasma concentrations of LTB4 are shown in T1D individuals sorted by the presence or absence of cardiac autonomic neuropathy (CAN) (adjusted for the same aforementioned confounders plus diabetes retinopathy, peripheral neuropathy, and diabetes kidney disease).

T1D participants with microvascular complications presented lower *MYD88* mRNA expression when compared to T1D participants without microvascular complications (*P* = 0.0008 after adjustment for sex, age, HbA_1_C, diabetes duration, and use of ACEI, ARB, and statin) (Figure 2(b)). No differences were observed in the mRNA expressions of *ALOX5* and *LTB4R* and also in plasma LTB4 concentrations after adjustment for those confounding factors (Figures 2(a), 2(c), and 2(d), respectively). When participants were sorted according to the presence or absence of each one of the chronic microvascular complications, higher LTB4 concentrations were found in participants with CAN versus without CAN (*P* = 0.005; Figure 2(e)) after adjustment for those confounding factors and for DR, DKD, and peripheral neuropathy.

**Figure 3:**
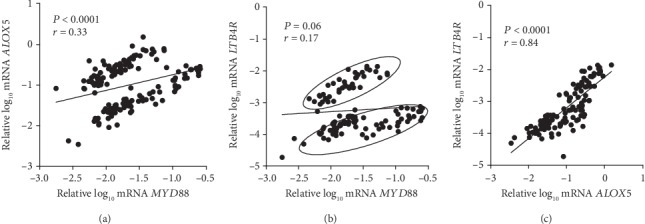
Correlations between mRNA expressions of (a) *ALOX5* and *MYD88*, (b) *LTB4R* and *MYD88*, and (c) *LTB4R* and *ALOX5* in peripheral blood mononuclear cells from type 1 diabetes individuals.

In T1D individuals, a positive correlation was observed between *MYD88* and *ALOX5* mRNA expressions (*r* = 0.33, *P* < 0.0001; Figure 3(a)), no correlation was observed between mRNA expressions of *MYD88* and *LTB4R* (Figure 3(b)), and a strong correlation was observed between *ALOX5* and *LTB4R* mRNA expressions (*r* = 0.84, *P* < 0.0001; Figure 3(c)).

The observation of two distinct subgroups of individuals in Figures 3(a) and 3(b) motivated us to analyze the characteristics of each one of these groups separately. As shown in Table 2, T1D participants sorted in Group B presented higher HbA_1_C, fructosamine, and plasma LTB4 concentrations and lower plasma GSH concentrations than those in Group A. There were no differences in the frequency of microvascular complications between these groups.

**Figure 4:**
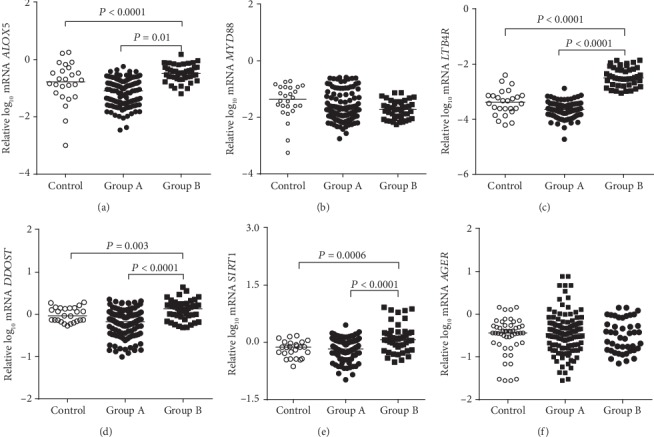
Expressions of *ALOX5*, *MYD88*, *LTB4R*, *DDOST*, *SIRT1*, and *AGER* mRNAs (a–f, respectively) in nondiabetic controls and in type 1 diabetes individuals sorted into Groups A and B (adjusted for sex, age, and use of angiotensin-converting enzyme inhibitor, angiotensin receptor blocker, and statin).

When individuals from Group A were compared to individuals from Group B and from nondiabetic controls, higher mRNA expressions of *ALOX5*, *LTB4R*, *DDOST*, and *SIRT1* were observed in Group B (Figures 4(a), 4(c), 4(d), and 4(e), respectively) after adjustment for sex, age, and use of ACEI, ARB, and statin. No differences were found in the mRNA expressions of *MYD88* and *AGER* (Figures 4(b) and 4(f), respectively). When only Group A and Group B were considered, the differences in the expressions of *ALOX5*, *LTB4R*, *DDOST*, and *SIRT1* mRNAs were maintained after adjustment for sex, age, diabetes duration, and use of ACEI, ARB, and statin (*P* < 0.0001 for all genes).

**Figure 5:**
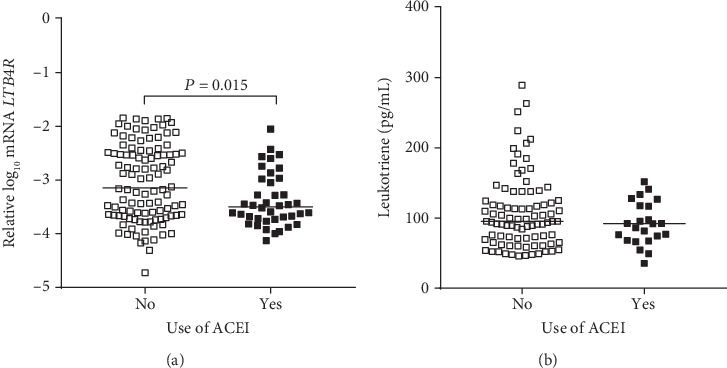
Expression of *LTB4R* mRNA in peripheral blood mononuclear cells (a) and plasma concentrations of leukotriene B4 (b) in type 1 diabetes individuals sorted by the use of angiotensin-converting enzyme inhibitor (ACEI) (adjusted for sex, age, HbA_1_C, diabetes duration, and use of angiotensin receptor blocker and statin).

We also investigated whether the mRNA expressions of *LTB4R*, *ALOX5*, and *MYD88* and the plasma concentrations of LTB4 would be modulated by ACEI, ARB, and statin; the mRNA expression of *LTB4R* was significantly lower in T1D individuals using ACEI after adjustment for sex, age, HbA_1_C, diabetes duration, and use of ARB and statin (*P* = 0.015; Figure 5(a)). Lower plasma concentrations of LTB4 were observed in T1D individuals using ACEI (91.9 pg/mL (68.2-117.1)) in comparison to individuals not using this medicine (95.3 pg/mL (69.9-134.5)), but this difference did not reach statistical significance (*P* = 0.07, Figure 5(b)).

## 4. Discussion

We evaluated the mRNA expressions of *ALOX5*, *LTB4R*, and *MYD88* in PBMC and the plasma concentrations of LTB4 in nondiabetic controls and in long-term T1D individuals. Besides observing higher expression of *LTB4R* in T1D individuals in comparison to nondiabetic controls, we found a lower expression of *MYD88* mRNA in individuals with complications in comparison to those without complications and higher concentrations of LTB4 in individuals with CAN. Additionally, we observed the modulation of the LT pathway by the degree of glycemic control.

*MYD88* encodes an essential cytosolic adapter protein that plays a central role in signal transduction of IL1 and of TLRs [17], besides participating in AGE signaling [18]. A lower expression of *MYD88* mRNA in patients with complications was an unexpected finding, especially because MYD88-dependent pathways were found to participate in the development of DR [19], and when we evaluated the expression of *MYD88* mRNA in patients sorted by microvascular complications, patients with DR presented a lower expression of this gene in comparison to patients without DR, with a *P* value of 0.06 (data not shown). However, the aforementioned study evaluated mouse retinal lysates and it is probable that gene expression in circulating cells do not reflect the intratissue condition. It is still intriguing that *MYD88* mRNA expression seems to be lower in PBMC from patients with DR and we do not have an explanation for this finding.

CAN is a neglected microvascular complication which increases the risk of cardiac arrhythmias, of atherosclerosis progression, and of sudden death [20]. The first abnormality usually observed in the course of CAN is an impairment of parasympathetic function, resulting in an imbalance of the sympathetic/parasympathetic tone that is followed by sympathetic denervation [21]. The finding of increased LTB4 concentration in patients with CAN suggests that the inflammatory response is partially controlled by the autonomic nervous system, but mechanistic studies are necessary to elucidate the relationship between the leukotriene pathway and dysautonomia. In the afferent arc, nerve sense injury and infection, and in the efferent arc, termed the cholinergic anti-inflammatory pathway, acetylcholine, the main parasympathetic neurotransmitter, exerts a tonic inhibitory role in the immune cells, comprising a reflex that limits proinflammatory responses within a nontoxic range [22]. Our data corroborate previous reports that autonomic dysfunction is associated with low-grade inflammation in T1D individuals, as evidenced by higher serum concentrations of C-reactive protein [23] and by a negative correlation between IL6 and the expiration : inspiration ratio of heart rate variability, which reflects the parasympathetic function [24].

The observation of two distinct subgroups in the mRNA expression correlation analyses led us to examine their characteristics. What differentiated the two groups was the degree of glycemic control; the group presenting higher expression of *ALOX5* and of *LTBR4* mRNAs also presented worst glycemic control. The activation of the LT pathway was confirmed by the higher concentrations of plasma LTB4 found in this group, even after adjustment for confounding variables, including the presence of CAN. These data are consistent with a study conducted in T1D individuals with poor glycemic control that showed increased production of LTB4 by polymorphonuclear leukocytes [25] and, together with the finding of higher *LTB4R* expression in T1D individuals in comparison to nondiabetic controls, reinforce the role of hyperglycemia in triggering sterile inflammation [12].

Interestingly, unlike *ALOX5* and *LTBR4*, *MYD88* expression was not modulated by hyperglycemia. Additionally, significant positive correlations were observed between *ALOX5* and *LTB4R* and between *ALOX5* and *MYD88*, but not between *LTB4R* and *MYD88*. These findings suggest that independent factors may control *MYD88* expression, and further studies are necessary to elucidate this issue.

Because MYD88 is one of the molecules involved in RAGE signaling, we also evaluated the mRNA expression of this AGE receptor, but no differences were observed in *MYD88* or *AGER* expression. The higher expressions of *DDOST* and *SIRT1* in the group presenting the worst metabolic control probably reflect a compensatory defense mechanism against the oxidative stress triggered by hyperglycemia, since *DDOST* encodes AGE-R1, a receptor involved in AGE clearance and in the activation of sirtuin-1, among other effects which ultimately restrict prooxidative and proinflammatory pathways [6].

Finally, the finding of lower expression of *LTB4R* in PBMC from T1D individuals receiving ACEI in comparison to those not taking them demonstrates one more pleiotropic anti-inflammatory effect of inhibitors of the renin-angiotensin system. ACEI were shown to inhibit LTA4 hydrolase (and consequently, LTB4 synthesis) [26], to reduce monocyte chemoattractant protein-1 expression in macrophages [27] and to decrease IL6 and tumor necrosis factor in normotensive type 2 diabetes individuals [28], among other effects.

## 5. Conclusion

In conclusion, in the diabetes setting, the LT pathway is not only activated by hyperglycemia but is also modulated by the status of the autonomic nervous system. The latter finding may contribute to the cardiovascular burden imposed by CAN, considering that studies *in vitro* and in animals suggest that LTB4 and other LT participate in the development of atherosclerosis [29].

## Figures and Tables

**Table 1:** Demographic, clinical, and biochemical characteristics of type 1 diabetes individuals.

	T1D participants (*n* = 164)
*Clinical and biochemical characteristics*	
Age (year)	34 (28–41)
Sex, female (%)	62
BMI (kg/m^2^)	24.4 (22–27.5)
eGFR (mL·min^−1^·1.73 m^2^)	90 (70-110)
Arterial hypertension (%)	15
Total cholesterol (mg·dL^−1^)	165 (147–185)
(mmol·L^−1^)	4.3 (3.8–4.8)
Triglycerides (mg·dL^−1^)	79 (56–103)
(mmol·L^−1^)	0.89 (0.63–1.16)
*Diabetes status*	
Diabetes duration (years)	20 (15–27)
Age at diagnosis (years)	12 (7–18)
HbA_1_C (%)	8.2 (7.3–9.4)
(mmol·mol^−1^)	66 (56–79)
Fructosamine (*μ*mol·L^−1^)	375 (321–450)
*Microvascular complications*	
Retinopathy (%)	69.5
Diabetic kidney disease (%)	29.7
Peripheral neuropathy (%)	40.1
Cardiovascular autonomic neuropathy (%)	46.7
*Use of medicines*	
ACEI (%)	28.2%
Statin (%)	38.9%

Data are expressed as median ± interquartile interval. BMI: body mass index; eGFR: estimated glomerular filtration rate; ACEI: angiotensin-converting enzyme inhibitors.

**Table 2:** Demographic, clinical, and biochemical characteristics of type 1 diabetes individuals sorted into the two subgroups shown in Figures 3(a) and 3(b).

	Type 1 diabetes patients		*P* value
	Group A (*n* = 120)	Group B (*n* = 44)	
*Clinical and biochemical characteristics*			
Age (year)	33 (22–42.7)	35 (26.7–39)	NS
Sex, female (%)	62.5	61.4	NS
BMI (kg/m^2^)	24.0 (21.9–26.8)	25.3 (22.8–27.9)	NS
eGFR (mL·min^−1^·1.73 m^2^)	90 (69.5–111)	92 (72–107)	NS
Arterial hypertension (%)	31.2	35.7	NS
Total cholesterol (mg·dL^−1^)	166 (147–185)	164 (147–187)	NS
(mmol·L^−1^)	4.3 (3.8–4.8)	4.2 (3.8–4.8)	
Triglycerides (mg·dL^−1^)	80 (56–109)	79 (56–93)	NS
(mmol·L^−1^)	0.9 (0.6–1.2)	0.89 (0.6–1.0)	
*Diabetes status*			
Diabetes duration (years)	20 (15–27)	20 (13.7–28)	NS
Age at diagnosis (years)	12 (8–19)	12 (6–17.5)	NS
HbA_1_C (%)	7.9 (7.1–9.3)	8.6 (8.1–9.8)	0.01
(mmol·mol^−1^)	63.5 (54.2–78.1)	71.1 (64.9–83.4)	
Fructosamine (*μ*mol·L^−1^)	369 (310–434)	408 (359–479)	0.02
*Microvascular complications*			
Retinopathy (%)	70.6	65.9	NS
Nephropathy (%)	30	27.3	NS
Peripheral neuropathy (%)	40	40.3	NS
Cardiovascular autonomic neuropathy (%)	43.3	54.5	NS
*Use of medicines*			
ACEI (%)	31.9	18.1	NS
Statin (%)	36.4	45.5	NS
*Oxidative markers*			
GSH (*μ*·nmol·mL^−1^)	1.05 (0.86–1.42)	0.88 (0.65–1.04)	<0.0001
TBARS (nmol·mL^−1^)	2.17 (1.41–6.18)	3.64 (2.20–4.90)	NS
LTB4 (mg·mL^−1^)	76.3 (60.2–105.7)	116.7 (95.3–139.3)	<0.0001

Data are expressed as median ± interquartile interval. ACEI: angiotensin-converting enzyme inhibitors; BMI: body mass index; eGFR: estimated glomerular filtration rate; GSH: reduced glutathione; TBARS: thiobarbituric acid-reactive substances.

## Data Availability

The data used to support the findings of this study are available from the corresponding author upon request.
